# The regulatory principles, physiological functions, and phase transition of biomolecular condensates

**DOI:** 10.3389/fcell.2026.1759561

**Published:** 2026-01-21

**Authors:** Qingxiang Li, Yan Jiang, Yan Chen

**Affiliations:** 1 Department of Pediatrics, Affiliated Hospital of Zunyi Medical University, Zunyi, China; 2 Guizhou Children’s Hospital, Zunyi, China

**Keywords:** biomolecular condensates, cellular organization, liquid–liquid phase separation (LLPS), molecular determinants, pathological phase transition, physicochemical regulation, post-translational modifications

## Abstract

Liquid-liquid phase separation (LLPS) is a crucial process that influences the spatial organization of cells. Dysregulation of this process can contribute to serious adverse outcomes, including neurodegenerative diseases, developmental disorders, and impaired immune responses. While various studies have explored how factors like physicochemical properties, molecular structures, and post-translational modifications (PTMs) affect the formation of condensates, current knowledge remains fragmented and lacks a cohesive framework. This review aims to systematically compile the principles regulating LLPS, focusing on the interplay between physico-chemical parameters, PTMs, and molecular sequence characteristics. Building on this foundation, we will also examine the significance of physiological phase separation and its connection to pathological phase transitions, such as the conversion from a liquid to a solid state.

## 1 Introduction

**FIGURE 1:**
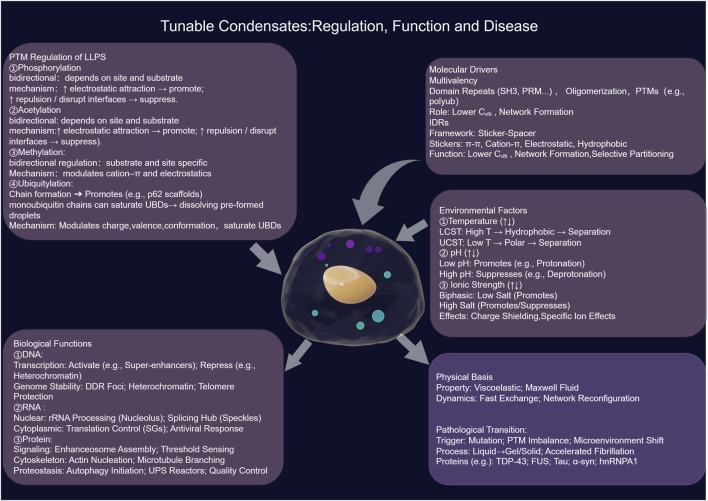
The regulation, assembly, and physiological and pathological functions of biomolecular condensates. This framework illustrates how biomolecular condensates, emerging from multivalent interactions and described by physical principles like the Flory-Huggins model, are tuned by the cellular environment, post-translational modifications (PTMs), and active interventions. This precise regulation enables their diverse functions in genome organization, RNA metabolism, and proteostasis. The dysregulation of this cycle—through mutation, imbalanced PTMs, or environmental stress—drives a pathogenic transition from dynamic liquids to solid-like aggregates, underpinning numerous human diseases.

Liquid–liquid phase separation (LLPS) has emerged as a transformative concept for elucidating subcellular compartmentalization through the dynamic formation of membrane-less organelles (Banani et al., 2017). Although the nucleolus was first identified as a liquid-like structure as early as 1898 (Montgomery, 1898) and early microscopic observations had documented the assembly of bio-macromolecular condensates (Montgomery, 1898), the molecular underpinnings of membrane-less organelles remained poorly understood until Brangwynne and colleagues revealed that P granules in *C. elegans* exhibit liquid-phase separation behavior (Brangwynne et al., 2009). In 2017, Banani et al. (2017) unified these diverse phenomena under the single, physicochemical concept of “Biomolecular Condensates.” By forming microenvironments with a specific composition and unique reaction kinetics, LLPS enables precise spatiotemporal control over biochemical reactions. This allows it to regulate diverse cellular processes such as mRNA metabolism, gene transcription, stress-responsive signaling, and protein quality control (Al-Husini et al., 2020; Liu et al., 2024; Protter and Parker, 2016; Sun et al., 2018; Zhou et al., 2019). For instance, microbial RNP condensates (BR-bodies) represent biomolecular condensates that concentrate RNA degradosome machinery. They act as platforms that enrich long, poorly translated mRNAs, sRNAs, and antisense RNAs to drive partial mRNA degradation, thereby preventing the buildup of toxic intermediates generated by endonucleolytic cleavage (Al-Husini et al., 2020). In addition,the ability of LLPS to compartmentalize transcriptional components also underlies its role in dynamic gene regulation. By concentrating coactivators and core machinery into biomolecular condensates, phase separation enables rapid and reversible control of transcription (Sabari et al., 2018). However, its dysregulation is also linked to severe pathological outcomes, including abnormal embryogenesis, immune dysfunction, and neurodegenerative protein aggregation (Lipiński et al., 2022; Meng et al., 2025; Qin et al., 2022). Mechanistically, LLPS is driven by a competition between intermolecular interactions and hydration. When attractive forces between molecules supersede their interactions with water, the system undergoes demixing, forming dilute and condensed phases (Kumaraswamy, 2017); The resulting condensates act as biochemical compartments that concentrate reactants and sharpen reaction thresholds (Goetz and Mahamid, 2020; Hyman and Simons, 2012). Despite significant progress over the past few decades in identifying diverse regulators of liquid-liquid phase separation (LLPS), the existing literature remains fragmented. There is a notable absence of comprehensive reviews that systematically integrate regulatory principles across different scales, encompassing physicochemical inputs, molecular features, and post-translational modifications. This fragmentation limits our ability to form a coherent model for the controlled assembly and functional modulation of condensates. To address this, our review synthesizes current knowledge through the physical principles of the Flory-Huggins model, with an overarching framework illustrated in Figure 1. We systematically examine how environmental conditions influence phase boundaries; how PTMs alter valency and charge distribution; and how sequence-encoded features govern selective partitioning and mixing behavior. These insights are then connected to functional roles in genome organization, RNA processing, stress response, and proteostasis. We also discuss the physiological regulation of condensate dynamics and how disease mutations disrupt normal phase behavior, thereby revealing potential opportunities for therapeutic intervention through multi-factor modulation.

## 2 Core determinants of bio-molecular condensates

### 2.1 Thermodynamic basis and physicochemical regulation of LLPS

Phase separation minimizes the free energy of a system by partitioning it into two distinct phases. Within the framework of Flory-Huggins theory, the stability of these phases is governed by the balance between the entropy of mixing and the enthalpy of interaction (Flory, 1942; Huggins, 1942; Hyman et al., 2014); The Flory-Huggins theory determines the thermodynamic behavior of a system through the χ parameter, which measures the net difference between solvent and intermolecular interactions. When χ is negative, affinity dominates and the system remains a homogeneous single phase; at χ = 0, the system behaves as an ideal mixture driven solely by entropy; when χ is positive, effective repulsion exists, and phase separation occurs when the concentration exceeds a critical saturation concentration Csat, forming a dilute phase Csat and a dense phase (c_dense) with distinct compositions, between which chemical potential and osmotic pressure equilibria are established (Mittag and Pappu, 2022; Zeng et al., 2020). Phase separation occurs when net attractive intermolecular forces (enthalpic contribution) exceed a critical threshold, thereby overcoming the entropic drive toward a homogeneous mixture (Flory, 1942; Huggins, 1942; Hyman et al., 2014). Determining if a condensate arises from phase separation hinges on satisfying specific conditions. First, a mesoscopic density transition, occurring at scales vastly larger than molecular sizes, must be evident. This is a prerequisite. To then confirm phase separation as the underlying mechanism and the condensate as a coherent thermodynamic phase, two pivotal questions must be addressed: (i) whether the internal density of core components markedly differs from the surroundings, and (ii) whether the observed density fluctuations, resulting from dynamic exchange, match the theoretical expectations based on molecular properties (Mittag and Pappu, 2022). Critically, key cellularly relevant conditions, namely, concentration, temperature, pH, and ionic strength, function as central governing elements that tune this energetic balance by effectively modulating the local χ parameter (Flory, 1942; Huggins, 1942; Hyman et al., 2014; Krainer et al., 2021).

#### 2.1.1 Concentration

The critical saturation concentration (Csat) serves as a key parameter in thermodynamic modeling of phase separation, quantitatively defining the concentration threshold required for this process to occur. At thermodynamic equilibrium (coexistence), the concentrations of the coexisting phases correspond to the conjugate points on the binodal boundary of the free energy curve, which arises from the interplay between intermolecular forces and solvation effects (Banani et al., 2017; Kumaraswamy, 2017). Physically, Csat represents the concentration of the dilute phase at this boundary. Its value is governed by the overall interaction strength within the system, as quantified by the Flory-Huggins parameter χ: stronger attractive interactions lead to a lower Csat. This relationship is further supported by studies using engineered intrinsically disordered proteins, where increased hydrophobicity significantly reduces the concentration required for phase separation (Dzuricky et al., 2020). Once the total concentration surpasses Csat, multivalent interactions drive rapid phase separation, resulting in a stable multiphase system; below this threshold, the system remains homogeneous (Alberti et al., 2019). Csat is not merely a passive observable but an actively encoded regulatory hub determined by molecular properties. Lin et al. identified Csat as a crucial quantitative threshold for phase separation, demonstrating that it is intrinsically encoded in the “molecular grammar” of sequence information and can be enhanced through multivalent interactions (Wang et al., 2018). Consistent with this, Dzuricky et al. (2020) showed that Csat can be rationally programmed *via* sequence design, enabling predictive control over condensate formation both *in vitro* and *in vivo* (Dzuricky et al., 2020). These findings suggest that biological systems achieve precise spatiotemporal control of phase separation by evolutionarily tuning Csat through sequence-specific interactions. Functionally, the sharp concentration dependence mediated by Csat enables switch-like regulation of cellular processes. For instance, Li et al. reported that N-WASPΔ-induced activation of the Arp2/3 complex exhibits a nonlinear, threshold response at approximately 1,000 nM (Li et al., 2012). This example underscores a fundamental principle of biomolecular condensates: their assembly and functionality depend on a tightly regulated concentration window, with Csat serving as the central determinant of this switch-like behavior.

#### 2.1.2 Temperature

Temperature represents a fundamental physical factor in the regulation of liquid–liquid phase separation (LLPS). The entropy-enthalpy trade-off directly dictates the resulting phase behavior. From a thermodynamic perspective, the mixing free energy ΔG = ΔH–TΔS shows that temperature (T) amplifies the entropic contribution (–TΔS) while also influencing the enthalpic term (ΔH), thereby capable of reversing the tendency for phase separation (Flory, 1942; Huggins, 1942; Hyman et al., 2014). Two classic temperature-dependent patterns emerge: lower critical solution temperature (LCST)-type phase separation occurs at elevated temperatures. The dominant mechanism is hydrophobic interaction, which is entropy-driven: higher temperatures enhance the disorder of water molecules, promoting the aggregation of hydrophobic groups to reduce interfacial area. This LCST behavior is exemplified in engineered systems; for instance, Dzuricky et al. (2020) designed artificial intrinsically disordered proteins (IDPs) that undergo LLPS only above a tunable LCST, demonstrating how sequence-encoded hydrophobicity can be harnessed to create thermoresponsive condensates. Upper critical solution temperature (UCST)-type phase separation takes place at lower temperatures. This is typically governed by enthalpy-driven interactions such as hydrogen bonding or aromatic stacking, which become favorable under cooler conditions and drive molecular association (Dzuricky et al., 2020; Dignon et al., 2019); This principle is reflected in biological systems. For example, the human heat shock factor 1 (HSF1) forms stress-induced nuclear condensates under heat shock that exhibit temperature sensitivity consistent with LCST-type regulation, illustrating how cells exploit thermally responsive phase separation for physiological adaptation (Ren et al., 2025). Notably, the study by Dzuricky et al. (2020) further showed that the LCST of synthetic condensates could be precisely tuned by varying the fraction of hydrophilic residues, offering a design strategy for engineering organelles with programmable thermal responsiveness. Moreover, temperature can drive not only monotonic phase transitions but also multistate or reentrant phase separation. In RNA–polycation assemblies, the mixture remains homogeneous at low temperatures due to electrostatic attraction, undergoes phase separation at intermediate temperatures driven by dehydration entropy, and redissolves at high temperatures as a consequence of ionic screening (Pullara et al., 2022). This multiphase behavior shows that temperature-driven transitions are not controlled by one simple mechanism. Instead, they result from a dynamic rebalancing of molecular interactions. As the temperature changes, the importance of electrostatic, hydrophobic, and hydration forces shifts. This sequential change in their relative weights creates the complex shapes seen in phase diagrams.

#### 2.1.3 pH

pH stands as a pivotal environmental cue for regulating liquid-liquid phase separation (LLPS), exerting its effects by precisely tuning the protonation states of ionizable amino acid residues. This alteration redistributes net molecular charges, modulates conformational dynamics, and ultimately reshapes the interaction landscapes that drive condensation. A canonical pattern observed across numerous systems is that mild acidification often promotes LLPS by neutralizing negative charges and exposing hydrophobic patches, whereas alkaline conditions tend to suppress it by enhancing electrostatic repulsion (Doshi et al., 2025; Srivastav et al., 2020). The mechanism is elegantly exemplified by histidine residues, which function as naturally evolved molecular switches due to their protonation sensitivity near physiological pH (pKa ∼6.0–7.0). Gradual protonation within the pH range of 6.0–8.0 can dramatically shift LLPS thresholds, effectively tuning the critical saturation concentration and the material properties of the resulting condensates (Jeon et al., 2025; Kim et al., 2024). This sensitivity operates not as a simple binary on/off switch, but rather as a continuous tuning mechanism, allowing cells to convert subtle pH changes into sharp, switch-like transitions in phase behavior. Notably, the relationship between pH and LLPS is not unidirectional but often constitutes a bidirectional regulatory loop. A compelling *in vivo* example is the nucleolus, where phase-separated condensates themselves act as proton buffers. They establish and maintain an intrinsic pH gradient between the fibrillar center/dense fibrillar component (FC/DFC) and the surrounding nucleoplasm. This spatially constrained, self-generated pH differential is not a passive byproduct but actively promotes selective molecular partitioning and directional trafficking (Adame-Arana et al., 2020). This reveals a profound feedback mechanism: phase-separated compartments can actively sculpt their local physicochemical environment, which in turn reinforces their composition and functional identity, positioning LLPS as an active driver of spatial and metabolic homeostasis.

#### 2.1.4 Ion

**FIGURE 2:**
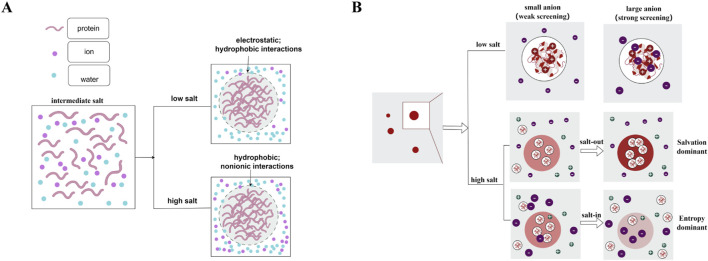
Regulation of phase separation by salt concentration and ion specificity. **(A)** The phase behavior of FUS is modulated by salt concentration: phase separation at low salt (mediated by electrostatic and hydrophobic interactions), dissolution at intermediate salt (due to electrostatic screening), and re-entrant phase separation at high salt (dominated by hydrophobic forces). The figure was adapted from the work of Krainer et al. (2021). **(B)** Ion-specific (Hofmeister) regulation mechanism: At low salt concentrations, small, strongly hydrated ions (e.g., Cl^−^) remain in the bulk solvent, while large, weakly hydrated ions (e.g., I^−^) approach the protein surface more closely, enhancing local electrostatic screening and promoting salting-out. At high salt concentrations, with electrostatics suppressed, phase behavior is governed by a competition between ion solvation energy and translational entropy. The figure was adapted from the work of Duan and Wang (2024).

Ions regulate protein phase separation dependent on the concentration and specific chemical properties, such as those described by the Hofmeister series. For example, Krainer and colleagues observed that the FUS protein phase-separates at low salt concentrations, forms a homogeneous solution at intermediate concentrations, and re-enters a phase-separated state at very high concentrations. The mechanism involves a shift from co-dominant electrostatic and hydrophobic interactions at low salt to a regime where high salt screens electrostatic forces, allowing hydrophobic and non-ionic interactions to dominate, which is accordance with Hofmeister series (as seen in Figure 2A) (Krainer et al., 2021). Additionally, ion size also critically influences phase behavior, even at a constant low concentration. Smaller ions like Cl^−^ tend to remain in the bulk solvent, whereas larger, weakly hydrated ions like I^−^ can approach the protein surface more closely. This proximity enhances local electrostatic screening, increasing their efficacy in promoting phase separation (salting-out). At high salt concentrations, where electrostatic interactions are fully suppressed, phase behavior is governed by a competition between ion solvation energy and translational entropy. Small, highly charged ions with strong hydration (e.g., Cl^−^, SO_4_^2−^) favor salting-out due to high solvation energy. Conversely, large, weakly hydrated ions with low charge density (e.g., SCN^−^, ClO_4_^−^) promote salting-in, driven by translational entropy and possible specific adsorption—behavior that aligns with the classic direct Hofmeister series (as seen in Figure 2B) (Duan and Wang, 2024). Moreover, beyond altering phase boundaries, ions can selectively partition into condensates to tune internal properties like interfacial tension and polarity (Zhu et al., 2024). In addition, specific ion binding such as Mg^2+^ or Zn^2+^ introduces a distinct layer of control. These ions coordinate with residues like histidines, acting as selective crosslinkers to stabilize or dissolve condensates independently of classical Hofmeister trends (Sołtys et al., 2023). Finally, such ionic perturbations carry functional and pathological relevance: for instance, they can drive liquid-to-solid transitions in disease-associated condensates (e.g., Tau protein aggregates), directly linking ion regulation to neurotoxic aggregation (Jonchhe et al., 2022). This underscores a direct mechanistic link between ion-specific regulation and disease-associated protein aggregation.

### 2.2 Structural and sequence features driving LLPS

While macroscopic parameters system-wide provide permissive conditions, the sequence-specific molecular determinants ultimately govern the propensity and properties of phase separation. This function is exclusive to a subset of proteins capable of physiological LLPS, conferred by multivalent interaction modules and/or IDRs.

#### 2.2.1 Multivalency-driven LLPS

Multivalency, enabled by repetitive folded domains (e.g., SH3, PDZ, coiled-coil) or short linear motifs (SLiMs; e.g., PRMs, RG/RGG), drives the formation of system-spanning interaction networks when the effective valency-affinity product exceeds a critical threshold. This network percolation shifts the phase boundary and lowers the saturation concentration (c_sat_),as exemplified in reconstituted systems like the Nephrin–NCK–N-WASP axis, where increasing SH3–PRM and SH2–pTyr interactions non-linearly promotes phase separation, consistent with percolation theory (Banani et al., 2017; Banani et al., 2016; Banjade and Rosen, 2014; Banjade et al., 2015; Li et al., 2012). Beyond intrinsic modularity, effective valence can be increased by oligomerization or by post-translational modifications that multiply binding sites. For example, G3BP1 dimerization *via* its NTF2-like domain expands RNA-binding capacity and strengthens RNA-driven phase separation (Yang et al., 2020), while pentamerization of NPM1’s N-terminal oligomerization domain arrays the A1/A2 acidic tracts to selectively engage partners bearing ≥2 arginine-rich R-motifs, thereby promoting condensation (Mitrea et al., 2016). However, this theoretical model often oversimplifies the dynamic complexity of biological systems, such as the neglect of cellular crowding effects and kinetic variables. Additionally, polyubiquitin chains encode tunable multivalency through linkage and length, engaging receptors such as p62 or RAD23B to assemble degradative condensates (Sun et al., 2018; Yasuda et al., 2020). This tunability of multivalency is not merely a mechanism for initiating phase separation, but more importantly, it provides the fundamental basis for organizing biomolecules within condensates according to their valence and interaction specificity. Consistent with this organizational principle, the scaffold–client model posits that multivalent scaffolds self-associate to build the structural and chemical core, while clients are recruited through unoccupied, physicochemically compatible sites on the scaffold (Banani et al., 2016).

#### 2.2.2 Intrinsically disordered regions regulated LLPS

##### 2.2.2.1 Molecular basis of IDR-driven phase separation

Intrinsically disordered regions (IDRs) are key building blocks of biomolecular condensates, yet *disorder per se* is insufficient to drive phase separation. IDRs function conforms to the sticker–spacer theory: modular sticker motifs mediate multivalent, weak interactions, while flexible spacers supply conformational entropy to sustain liquid-like dynamics (Martin et al., 2020). This behavior is encoded by low-complexity sequences (LCRs) enriched for charged (R/K; D/E), polar (G/Q/N/S), and aromatic (Y/F) residues that frequently appear as short repeats (e.g., YG/S-, RG-, SY), furnishing targets for multivalent contacts (Nott et al., 2015; Ravindran et al., 2023).

##### 2.2.2.2 IDR sequence-encoded regulation of condensate dynamics

IDRs do not rely on a single force; instead, they deploy a portfolio of weak, short-range interactions—electrostatic, hydrophobic, cation–π, π–π/sp^2^–π, and hydrogen bonding—that act in parallel to nucleate and stabilize condensates (Das et al., 2020; Murthy et al., 2021; Nott et al., 2015; Qamar et al., 2018). FUS exemplifies IDR-driven LLPS: its N-terminal SYGQ low-complexity segment, central RRM/zinc-finger domains, and C-terminal RGG repeats cooperate to promote condensation. [G/S]Y [G/S] motifs drive liquid-to-gel/solid transitions, while cation–π and hydrophobic interactions lower Csat. The RGG–SYGQ network further stabilizes droplets *via* hydrogen bonds, sp^2^–π stacking, and enhanced cation–π contacts (Kato et al., 2012; Murray et al., 2017; Murthy et al., 2021; Qamar et al., 2018; Wang and Li, 2024). These multimodal interactions endow FUS condensates with robustness and plasticity, yet their imbalance can drive pathological solidification. The functional spectrum of IDRs is highly diverse. For instance, in G3BP1, IDR1 acts as an autoinhibitory module that suppresses phase separation through intramolecular interactions, whereas IDR2 counteracts this inhibition to promote phase separation, collectively revealing a bidirectional control mechanism (Yang et al., 2020). Thus, the intricate modularity and regulatability of IDRs offer a promising foundation for developing targeted therapeutic interventions against condensation-associated pathologies.

##### 2.2.2.3 Selective organization and partitioning rules of IDRs

Coexisting condensates in cells always show spatial exclusivity (De La Cruz et al., 2024; King et al., 2024),reflecting sequence-encoded selectivity. In the nucleolus, IDR features (e.g., D/E tracts vs. K-blocks with E-rich segments) and associated RNA/DNA-binding domains bias partitioning among FC/DFC (King et al., 2024). Moreover, experimental swap assays further demonstrate this principle: exchanging MED1’s charged IDR features with glutamine/tyrosine motifs redirected proteins toward FUS condensates, whereas the reciprocal replacement recruited proteins to MED1 condensates (De La Cruz et al., 2024). Another example comes from the distribution of charge and aromatic residues, where dense RGG clusters promote homotypic separation and exclusivity, while dispersed charges favor heterotypic mixing (Jo et al., 2022; Welles et al., 2024). These findings provide a theoretical foundation for modulating and targeting phase-separated condensates. However, the predictive capacity of sequence features for partitioning behavior is constrained by incomplete understanding of multiscale interactions; and the dynamic interplay between IDRs and other cellular components remains underexplored.

### 2.3 PTM-tunable valence and affinity to regulate LLPS

The variable and disordered nature of the IDRs introduces a dynamic dimension for regulation, as their exposed modification sites may serve as prime targets for PTMs, thereby potentially fine-tuning phase separation (Clifford et al., 2015).

#### 2.3.1 Phosphorylation

Phosphorylation exemplifies the bidirectional nature of PTM regulation. By introducing negative charges, it can increase anionic character and enhance electrostatic attraction, thereby lowering Csat and facilitating droplet assembly (Tsang et al., 2019; Wegmann et al., 2018). Moreover, such effects are frequently threshold-dependent, with over-phosphorylation predisposing proteins toward pathological aggregation (Wegmann et al., 2018). In particular, phosphorylation can enhance condensate stability by stabilizing oligomeric conformations, as seen with S16 phosphorylation reinforcing the dimer structure of C/EBPα (Wang et al., 2023). Conversely, excessive or misplaced phosphorylation may raise repulsion or disrupt critical interfaces, attenuating LLPS. For instance, serine phosphorylation in FUS-LC and G3BP1 weakens phase separation (Monahan et al., 2017; Yang et al., 2020),while S54/S61 phosphorylation in FUS induces a β-to-α transition that suppresses both condensation and amyloidogenesis (Lao et al., 2022). This inherent capacity for precise, reversible, and context-dependent regulation makes phosphorylation an ideal molecular switch for controlling biomolecular condensate dynamics under physiological conditions. This concept is elegantly demonstrated in the regulation of both P granules in *C. elegans* and synaptic vesicle clusters in neurons, illustrating its role in driving both slow developmental transitions and fast physiological responses across evolutionarily distant systems (Milovanovic et al., 2018; Wang et al., 2014). The significant differences observed in these studies emphasize that the functional outcome is not solely dependent on the post-translational modification (PTM) itself; rather, it is intricately influenced by the specific site, local sequence, and structural context.

#### 2.3.2 Acetylation

Acetylation generally restrains LLPS through neutralizing lysine charge and weakens the electrostatic interactions that drive condensation, as observed in hyper-acetylated IRF3/IRF7 complexes or the SARS-CoV-2 nucleocapsid protein (Qin et al., 2022; Wang S. et al., 2021). Conversely, acetylation can promote condensate assembly when it stabilizes key scaffolding proteins; for instance, K276 acetylation on RB1CC1 prolongs scaffold availability and enhances autophagy-related phase separation (Yi et al., 2023). Notably, the functional outcome of acetylation is also determined by site and sequence context, as demonstrated in tau protein where acetylation at K311 promotes LLPS while K369 acetylation suppresses it (Powell et al., 2024). This site-specific mechanism, which parallels the findings in phosphorylation, enables precise and bidirectional regulation of LLPS, allowing a single post-translational modification to orchestrate distinct cellular processes.

#### 2.3.3 Methylation

Methylation further illustrates this duality by reprogramming interactome networks to dictate condensate state and function, primarily through modulating interaction strength and specificity. On one hand, hypomethylation of arginine residues strengthens Arg–Tyr cation–π interactions, driving aberrant gel-like or β-sheet-rich states as observed in FUS or MSX1 (Meng et al., 2025; Qamar et al., 2018). On the other hand, asymmetric dimethylation of FUS weakens homotypic LLPS but creates docking sites for SMN Tudor domains, establishing heterotypic FUS–SMN–RNA droplets with normal RNP function (Wang and Li, 2024). Extending beyond proteins, m^6^A RNA methylation increases transcript valency to nucleate selective condensates enriched with YTHDF proteins, which in turn regulate downstream processes such as mRNA fate and tumour suppression (Zhao et al., 2024).

#### 2.3.4 Ubiquitylation

Ubiquitylation provides a particularly versatile example of programmable multivalency and thereby gate condensate assembly (Du et al., 2022; Sun et al., 2018; Yasuda et al., 2020). Accordingly, in protein quality control, K48-linked tetraUb chains direct the assembly of degradation condensates by recruiting clients like RAD23B and proteasomal subunits (Yasuda et al., 2020). In contrast, K63-linked chains drive the formation of autophagic condensates through p62 oligomerization (Sun et al., 2018). Beyond degradation, these same K63 linkages can also nucleate NEMO condensates to activate IKK and NF-κB signaling (Du et al., 2022). Conversely, monoubiquitin or short/sparsely distributed chains can saturate UBDs without crosslinking, capping IDR-based hubs and dissolving pre-formed droplets (Dao et al., 2018). Similar to ubiquitination, the SUMO-ubiquitin cascade represents a central mechanism for protein stability control, as demonstrated in PML nuclear bodies where stress-triggered SUMO2/3 modification directly templates ubiquitination to orchestrate stem cell fate (Sarah et al., 2022).

The interplay between PTM type, specific site, and cellular context dictates diverse phase separation outcomes, as synthetically summarized in Table 1.

**TABLE 1:** Summary of how post-translational modifications (PTMs) bidirectionally regulate liquid-liquid phase separation (LLPS).

PTM	Regulation	Mechanism	Example	References
Phosphorylation	Positive	Introduces negative charge, enhances electrostatic attraction, stabilizes multivalent-competent conformations.	S16 phosphorylation stabilizes C/EBPα dimers, enhancing condensate stability	Wegmann et al. (2018), Tsang et al. (2019), Wang et al. (2023)
	Negative	Introduces excessive negative charge causing electrostatic repulsion; disrupts critical multivalent interaction interfaces.	Ser phosphorylation in FUS and G3BP1 disrupts homotypic LLPS, mitigating pathological aggregation	Lao et al. (2022), Monahan et al. (2017), Yang et al. (2020)
Acetylation	Positive	Neutralizes positive charge to reduce electrostatic repulsion; stabilizes scaffolding proteins to prolong condensate lifetime.	K276 acetylation on RB1CC1/FIP200 amplifies autophagy initiation by stabilizing pro-autophagic condensates.	Powell et al. (2024), Yi et al. (2023)
	Negative	Neutralizes lysine charge, weakening crucial cationic-π and other electrostatic driving forces for LLPS	Hyper-acetylation of IRF3/IRF7 impedes antiviral signalosome formation; observed in viral immune evasion.	Powell et al. (2024), Qin et al. (2022), Wang S. et al. (2021)
Arginine Methylation	Positive	Creates specific recognition codes for heterotypic interactions (e.g., Tudor domains); increases RNA valency.	FUS asymmetric dimethylation recruits SMN, forming functional RNP granules. m^6^A mRNA recruits YTHDFs into regulatory condensates.	Wang and Li (2024), Zhao et al. (2024)
	Negative	Hypomethylation strengthens non-specific cation-π interactions, driving aberrant phase transitions to gels/solids.	FUS hypomethylation promotes age-related pathological solidification in ALS models.	Meng et al. (2025), Qamar et al. (2018)
Ubiquitylation	Positive	Provides programmable multivalency (K63 chains) for scaffolding; acts as a recruitment signal (K48 chains) for functional complexes.	K63-linked ubiquitin nucleates NF-κB signalosomes *via* NEMO. K48 chains concentrate substrates in proteolytic condensates.	Du et al. (2022), Sun et al. (2018), Yasuda et al. (2020)
	Negative	Monoubiquitylation or short chains act as a dominant-negative by saturating UBDs and capping multivalent interactions.	Monoubiquitin dissolves pre-formed condensates by sequestering ubiquitin-binding proteins (e.g., hHR23B).	Dao et al. (2018)

The table categorizes representative PTMs, by their general mechanisms of altering molecular valence or affinity, their functional outcomes in promoting or inhibiting LLPS, and key examples. The effects are highly dependent on the specific modification site, type, and cellular context. PTM, post-translational modification; Ub, ubiquitin.

### 2.4 The regulatory roles of RNA and molecular crowding in LLPS

As one of the most abundant multivalent macromolecules in the cell, RNA acts not only as a “client” but also as a key regulator of phase separation, dynamically modulating its occurrence, material properties, and biological functions through diverse mechanisms (Patel et al., 2015; Zhang et al., 2015). Specifically, RNA exhibits a notable concentration-dependent biphasic effect on phase separation. At lower concentrations, RNA can promote phase separation by serving as a molecular bridge *via* nonspecific electrostatic interactions between its negatively charged backbone and positively charged intrinsically disordered regions (IDRs) of proteins. In contrast, at higher concentrations, excess RNA molecules compete for binding interfaces on proteins, disrupting the multivalent interaction network and thereby inhibiting phase separation or even dissolving preformed condensates. This re-entrant phase behavior underscores that RNA concentration is a central determinant of the dynamics and state of biomolecular condensates (Patel et al., 2015; Zhang et al., 2015). Beyond concentration, the sequence and structural specificity of RNA also contribute to the regulation of liquid–liquid phase separation (LLPS). For instance, enhancer RNAs (eRNAs) and long non-coding RNAs (lncRNAs) can engage in precise molecular recognition with specific proteins, enabling spatially defined recruitment of components and guiding the assembly of functionally distinct biomolecular condensates (Sabari et al., 2018). However, the crowding agents such as PEG are commonly used to mimic the crowded intracellular environment and reconstitute phase separation *in vitro*, high concentrations of inert polymeric crowders may non-specifically induce phase separation or drive the formation of poorly dynamic assemblies-phenomena that may not occur or be physiologically relevant under native cellular conditions (Mittag and Pappu, 2022).

### 2.5 Condensates as functional modules in cellular organization

Given that biomolecular condensates are dynamically and precisely regulated, a critical question arises: what specific cellular functions do they organize? This organization governs a wide range of biological processes, from gene expression to proteostasis.

#### 2.5.1 DNA-associated functions of LLPS

##### 2.5.1.1 LLPS in genomic stability

Liquid-liquid phase separation (LLPS) stabilizes the genome by modulating key processes such as DNA damage signaling, heterochromatin organization, telomere maintenance and assembly of critical cellular structures. While studies show that condensates like TopBP1 amplify ATR–Chk1 signaling and stabilize replication forks (Frattini et al., 2021); and that 53BP1 compartments modulate repair pathway choice (Zhang et al., 2022),a crucial question remains: how do these hubs prevent spurious activation? The enormous capacity for amplification could potentially lead to excessive signaling from minor lesions. The finding that SLX4-SUMO condensates regulate the timely resolution of repair intermediates (Alghoul et al., 2023) hints at built-in termination mechanisms. At telomeres, shelterin components such as TRF1 and TRF2 facilitate the formation of telomric condensates that promote higher-order compaction and T-loop formation. Furthermore, the role of LLPS extends to the assembly of critical cellular structures. A prime example occurs during mitosis, where the phase separation of NuMA, regulated by Aurora A kinase, drives the precise assembly of the mitotic spindle (Mengjie et al., 2021). In heterochromatin maintenance, LLPS driven by HP1 proteins, reinforced by H3K9me3 deposition and ATRX–DAXX–mediated H3.3 incorporation, facilitates the compaction and functional partitioning of heterochromatic regions. Notably, 53BP1–HP1α condensates contribute to heterochromatin stability largely independent of canonical double-strand break repair, suggesting a structural role in genome organization beyond acute damage handling (Larson et al., 2017; Zhang et al., 2022). These compartments function not only structurally but also protectively by limiting spurious engagement of DNA damage response factors, thereby preventing illegitimate repair and preserving chromosome ends (Jack et al., 2022). This critical perspective suggests that LLPS functions as a double-edged sword in heterochromatin, balancing compaction with accessibility, a tension that is not yet fully explored. Notably, aberrant transcriptional condensates can drive tumorigenesis by inducing chromosomal structural abnormalities. For instance, in leukemia, the aberrant transcriptional condensates formed by the NUP98-HOXA9 fusion protein orchestrate tumorigenesis through a dual mechanism: mimicking super-enhancer functionality to hyperactivate proto-oncogenes while concurrently remodeling the 3D chromatin architecture through anomalous looping, ultimately leading to uncontrolled cellular proliferation (Jeong Hyun et al., 2021).

##### 2.5.1.2 LLPS in gene transcription regulation

Liquid-liquid phase separation (LLPS) has emerged as a pivotal mechanism in gene transcription regulation, functioning as a bidirectional switch that dynamically assembles membrane-less compartments to control genomic output. On the activating front, coactivators like BRD4 and Mediator form condensates at super-enhancers that enrich the transcriptional machinery, facilitating Pol II initiation and productive elongation, with Pol II-CTD modifications further tuning engagement within these hubs to promote promoter escape (Boehning et al., 2018; Boija et al., 2018; Sabari et al., 2018). Conversely, repressive condensates regulate gene silencing by compacting chromatin and reducing its accessibility. Polycomb assemblies (e.g., PRC1/CBX2) undergo phase separation to achieve acute compaction, which is then stabilized by sustained H3K27me3 deposition to maintain long-term repression. Additionally HP1α droplets nucleate at H3K9me3 sites to reinforce heterochromatin organization (Larson et al., 2017; Plys et al., 2019). Beyond the behavior of individual protein complexes, the material state of chromatin itself is increasingly recognized as an active participant in this process; the condensation of nucleosome arrays, driven by histone tails or modulated by small molecules like Ru1, alters viscoelasticity, reconfigures enhancer-promoter communication, and ultimately shifts transcriptional output (Gibson et al., 2019; W. J. Wang et al., 2021). These findings offer more dynamic perspective than traditional static models for understanding chromatin reorganization. Future efforts should focus on the integration of LLPS biophysics with mechanistic studies of transcriptional control.

#### 2.5.2 RNA associated phase separation

RNA-related membrane-less organelles, such as the nucleolus, nuclear speckles, and stress granules, serve as prime models for liquid-liquid phase separation (LLPS). These condensates assemble reversibly *via* multivalent weak interactions at physiological concentrations, coupling processes like transcription, splicing, translational repression, and quality control, while enabling rapid molecular reallocation under stress (Brangwynne et al., 2009; Feric et al., 2016; Lafontaine et al., 2021; Mitrea et al., 2018; Yang et al., 2020).

##### 2.5.2.1 LLPS as an organizer of dynamic RNP organelles

Despite distinct compositions, major RNA–protein condensates share a common assembly principle: multivalent interactions mediated by intrinsically disordered regions (IDRs) and RNA initiate core formation, while interfacial energy differences drive multiphase organization. For instance, the nucleolus nucleates *via* high rRNA transcription, organizing into a tripartite condensate (FC, DFC, GC) scaffolded by electrostatic/π-interaction networks involving NPM1, rRNA, and arginine-rich proteins like SURF6; compartmentalization is refined by interfacial tension, facilitating ordered rRNA processing (Feric et al., 2016; Ferrolino et al., 2018; Lafontaine et al., 2021; Mitrea et al., 2018). Similarly, nuclear speckles form through LLPS driven by IDR-rich scaffolds like SON and SRRM2, with phosphorylation dynamically regulating architecture (Ilik et al., 2020; Prashant et al., 2024; Xu et al., 2022). In contrast, stress granules (SGs) assemble *via* multi-step condensation of untranslated mRNPs, stabilized by G3BP1, CAPRIN1, and TIA1 networks (Jain et al., 2016; Wheeler et al., 2016; Yang et al., 2020).

##### 2.5.2.2 RNA condensates as functional nodes in cellular metabolism and stress adaptation

Functionally, RNA condensates modulate cellular metabolism and stress responses through the selective concentration and release of RNAs and ribonucleoproteins (RNPs). In the nucleus, the nucleolus exemplifies this principle by forming a biomolecular condensate that provides a viscoelastic environment enhancing pre-rRNA processing efficiency (Feric et al., 2016; Lafontaine et al., 2021). Under stress conditions, cells employ phase separation to reconfigure the splicing machinery, precisely tuning mRNA output through widespread intron retention. A key illustration of this is the CLK1/2 kinase–driven remodeling of nuclear speckles, which promotes intron retention to fine-tune mRNA output (Prashant et al., 2024; Wang B. et al., 2024; Xu et al., 2022), highlighting how phase separation integrates transcriptional and post-transcriptional control. In the cytoplasm, stress granules (SGs) transiently sequester mRNAs to arrest translation. DYRK3-mediated dissolution of SGs enables translational recovery upon stress alleviation (Jeon et al., 2024). However, the functional implications of SGs remain context-dependent, as persistent SGs do not always correlate with cellular rescue, suggesting complex regulation beyond mere condensation. At a systems level, RNA concentration bidirectionally regulates condensates: low levels stabilize *via* electrostatic complementarity to enhance transcription, while high levels dissolve them (Henninger et al., 2021). Beyond translational control, emerging evidence indicates that SGs act as immunomodulatory hubs by linking translational arrest to innate immune signaling. For instance, TRIM25–G3BP1 co-condensation facilitates the tuning of the RIG-I/MDA5 pathway (Onomoto et al., 2014; Shang et al., 2024). Nevertheless, the specificity of immune modulation through phase separation is debated, particularly in viral infections, where pathogens may exploit LLPS to evade immune detection.

#### 2.5.3 Protein associated LLPS

Protein-associated LLPS generates membrane-less condensates that enrich reactants and tune kinetics, thereby organizing signaling, cytoskeletal dynamics, and proteostasis.

##### 2.5.3.1 LLPS in signal transduction

Membrane-less condensates establish high-activity reaction hubs that amplify signaling outputs and sharpen response thresholds. At the plasma membrane, multivalent receptor–adaptor assemblies (e.g., EGFR–Grb2) exhibit condensate-like properties that potentiate downstream activity, suggesting a general mechanism for signal amplification (Lin et al., 2022). This principle extends to the nucleus, where YAP-derived condensates enrich transcription factors like TEAD1 to drive proliferative programs (Cai et al., 2019). Innate immune responses are similarly regulated through opposing LLPS modalities: while polyubiquitin-driven condensation of NEMO amplifies NF-κB signaling, STING forms inhibitory condensates that prevent hyperactivation (Du et al., 2022; Yu et al., 2021). These findings reveal that LLPS enables bidirectional control of signaling pathways. However, a key limitation across these studies is the heavy reliance on overexpression systems and engineered constructs, leaving open the question of whether such condensates form at endogenous expression levels and under physiological conditions. This gap is particularly relevant in disease contexts: although LLPS-enabled super-enhancers (e.g., containing LSD1, BRD4, and FOXA1) sustain oncogenic signaling in castration-resistant prostate cancer (Li et al., 2023), and aberrant glycogen phase separation promotes hepatocarcinogenesis *via* YAP activation (Liu et al., 2021), the direct causal role of phase separation in tumorigenesis, *versus* its role as a correlative epiphenomenon, remains inadequately tested. Thus, while IDR-driven phase separation is proposed to constitute a central layer of signaling control, the field must critically evaluate its mechanistic necessity *in vivo*.

##### 2.5.3.2 LLPS in the cytoskeleton

LLPS directly orchestrates the spatial organization of the cytoskeleton by unifying force-producing components. For instance, reconstituted PSD condensates promote F-actin bundling and spine growth through charged surface interactions on Homer EVH1 (Chen et al., 2023). This finding is complemented by studies on abLIM1, whose condensates integrate actin nucleation and crosslinking to assemble tension-bearing cortical networks under low-salt conditions (Sen et al., 2022). A parallel logic applies to microtubule regulation: TPX2–tubulin co-condensation enhances branching nucleation on pre-existing microtubules, while importin-α/β suppresses condensation to impose spatiotemporal precision (King and Petry, 2020). Collectively, these examples illustrate how phase separation links upstream signals to the architectural programming of the cytoskeleton. This mechanism paves the way for innovative drug development targeting major diseases such as cancer metastasis and neurodegenerative disorders and facilitates the creation of novel smart biomaterials.

##### 2.5.3.3 LLPS in proteostasis

LLPS plays a dual role in proteostasis, facilitating both the targeted degradation of ubiquitinated substrates and the formation of adaptive stress-responsive compartments. p62/SQSTM1 undergoes K63-ubiquitin–driven phase separation to initiate selective autophagy (Sun et al., 2018), while in the nucleus, p62 condensates function as proteasomal “reactors” that enrich K48-ubiquitylated substrates to accelerate degradation (Yasuda et al., 2020). Under stress, the proteasome itself forms reversible nuclear condensates (SIPANs) that co-assemble substrates, receptors, and machinery into a single functional unit (Uriarte et al., 2021). Similarly, RNP granules such as P-bodies and stress granules triage misfolded clients and silenced mRNPs, with their dysfunction leading to proteotoxic stress (Nostramo et al., 2019). The material properties of these granules are actively maintained by chaperone systems like HSPB8–BAG3–HSP70, which prevent pathological solidification (Ganassi et al., 2016); and regulated by factors such as NS1-BP, which stabilizes stress granules to enable their autophagic clearance (Jeon et al., 2024). These studies collectively position LLPS as a central organizer of protein quality control. However, they also reveal a critical, unresolved tension: the same physicochemical forces that promote adaptive compartmentalization can, under dysregulated conditions, drive a liquid-to-solid transition that underlies neurodegenerative pathology. This underscores the need to better understand the physiological checks and balances that maintain functional phase separation and prevent its collapse into disease-associated aggregates.

**FIGURE 3:**
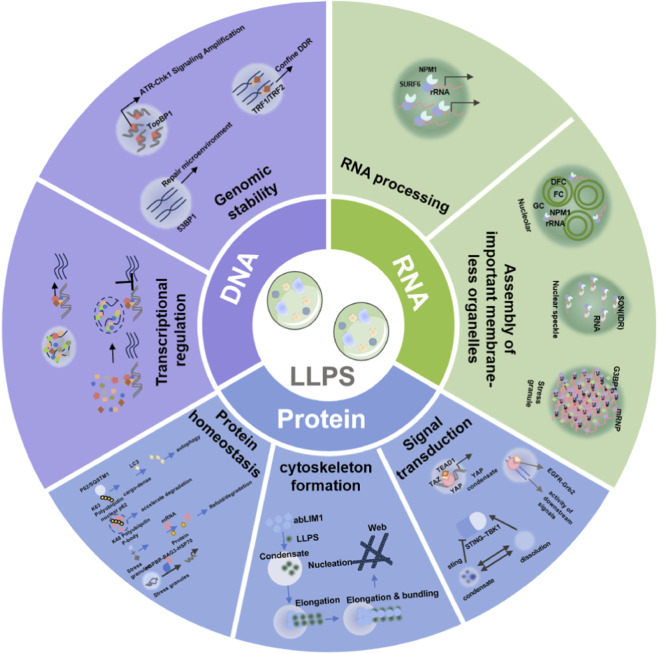
Schematic model depicting the organization of key cellular processes by biomolecular condensates *via* liquid-liquid phase separation (LLPS). DNA associated regulation:LLPS stabilizes heterochromatin and facilitates DNA repair. In addition, LLPS bidirectionally controls transcription through forming activating hubs at super-enhancers or repressive hubs for gene silencing. RNA associated regulation: Liquid-liquid phase separation (LLPS) mediates the assembly of dynamic ribonucleoprotein (RNP) organelles (nucleolus, nuclear speckles, cytoplasmic stress granules). Protein associated regulation: LLPS coordinates signaling, cytoskeleton, and proteostasis.

LLPS serves as a universal organizing principle across the central dogma, coordinating genome stability, transcriptional regulation, dynamic RNP organelle assembly, and protein homeostasis, as integrated in Figure 3.

#### 2.5.4 From liquid to solid: Physiological spectra and pathological transformations

The mechanisms governing condensate formation and function converge on a critical question: what are the physiological characteristics of these compartments? Understanding the spectrum of their material states—from liquid to solid—and their physiological transitions is paramount, as dysregulation of these dynamics represents a cornerstone of disease pathogenesis.

##### 2.5.4.1 Physical basis and regulation of LLPS phase transitions

On short timescales (t < τ_relax), biomolecular condensates predominantly display elastic responses; over longer periods (t > τ_relax), viscous flow becomes dominant. This macroscopic behavior can be provisionally captured by classical Maxwell-type viscoelastic models. Importantly, condensate “aging” involves more than a simple rise in viscosity. Research indicates that aging is principally driven by time-dependent internal network remodeling, governed by sequence-specific intermolecular interactions—such as hydrogen bonds and π–π stacking—that function as physical crosslinks (Alshareedah et al., 2024). These interactions directly strengthen network connectivity and elevate the elastic modulus (G′), rather than merely increasing macroscopic viscosity. Consequently, aging manifests as a marked extension of the relaxation time (τ_relax), slowed fusion and rounding dynamics, and systematic shifts in rheological moduli as well as fluorescence recovery after photobleaching (FRAP) kinetics (Jawerth et al., 2020; Mukherjee et al., 2024); Such network remodeling has direct functional repercussions. Aging can reduce molecular exchange rates and substrate accessibility within condensates, leading to time-dependent declines or alterations in biochemical activities, including catalytic function (Kang et al., 2025). This indicates that the balance between stability and dynamics, maintained through metastable free-energy landscapes and multivalent interactions, is inherently dynamic and precarious. Notably, aging may progress toward irreversible transitions. Under pathological conditions, liquid-liquid phase separation (LLPS) itself can serve as a platform for aberrant aggregation. For example, α-synuclein undergoes LLPS to form condensates that dramatically concentrate the protein, creating a local environment conducive to irreversible conformational changes and drives the irreversible solidification of liquid condensates into amyloid fibrillar aggregates (Ray et al., 2020). Beyond internal interactions and aging, condensate phase behavior is modulated by a spectrum of factors including interfacial effects, viscoelastic relaxation, the competition between nucleation and spinodal decomposition pathways, local crowding, and chaperone activity (Visser et al., 2024).

##### 2.5.4.2 Phase transition of LLPS

**FIGURE 4:**
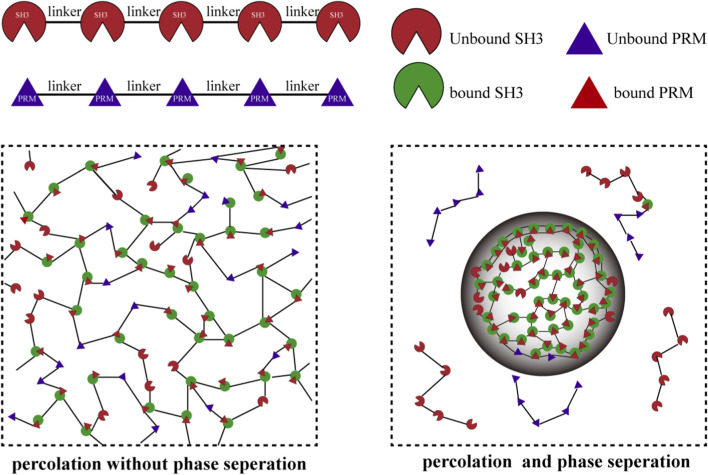
The sticker–spacer framework and the coupling of phase separation to percolation. Multivalent interactions between repeated “sticker” domains (e.g., SH3 and PRMs) drive a percolation transition above a threshold concentration, c_perc. Linker-dependent solubility dictates the outcome: if the saturation concentration Csat < c_perc (right), percolation couples with phase separation; if c_perc < Csat (left), a percolated network forms without demixing. (Adapted from Harmon et al., 2017). Abbreviations: LLPS, liquid-liquid phase separation; RNP, ribonucleoprotein; SG, stress granule; IDR, intrinsically disordered region; Pol II, RNA polymerase II; CTD, C-terminal domain; H3K9me3, histone H3 lysine 9 trimethylation; rRNA, ribosomal RNA; mRNA, messenger RNA; mRNP, messenger ribonucleoprotein; F-actin, filamentous actin.

Proteins and RNA can drive phase transitions through multivalent interaction modules. These typically consist of sticker motifs, such as aromatic or arginine residues, and flexible or disordered spacer linkers. The sticker–spacer framework proposes that reversible associations between stickers can drive two distinct yet often coupled transitions: liquid–liquid phase separation, governed by net attraction as quantified by a renormalized chi parameter and occurring above a saturation concentration (Csat); and a percolation transition, driven by network connectivity and defined by a percolation threshold (c_perc), which leads to the formation of a system spanning physical gel. When Csat is lower than c_perc, and c_perc in turn lies below the concentration in the dense phase, phase separation and percolation become tightly coupled. The resulting condensates thus form reversible physical microgels that exhibit time dependent viscoelastic properties rather than irreversible solidification (as seen in Figure 4) (Choi et al., 2020; Harmon et al., 2017; Mittag and Pappu, 2022). Since 2015, studies have revealed that the pathological aggregation of many neurodegenerative disease related proteins, such as tau, α synuclein, and TDP 43, is closely associated with phase transitions (Cao et al., 2019; Fitzpatrick et al., 2017; Li et al., 2018). These proteins can form fibrils through two primary nucleation pathways: classical nucleation in homogeneous solution (percolation transition), and condensate mediated nucleation. The latter is governed by a two threshold model in which liquid–liquid phase separation (LLPS) is initiated at a lower concentration, generating protein rich condensates. A subsequent, higher concentration threshold within these condensates then triggers the liquid to solid transition, dramatically accelerating fibril formation (Mathieu et al., 2020; Molliex et al., 2015; Patel et al., 2015; Ray et al., 2020; Wegmann et al., 2018). Notably, biomolecular condensates can exert dual regulatory effects on nucleation. For example, full length α synuclein, due to its amphipathic nature, accumulates at condensate interfaces where elevated local concentrations reduce the nucleation energy barrier and promote fibrillation. In contrast, truncated variants of α synuclein can experience nucleation suppression within certain condensates, likely due to stabilizing molecular interactions (Lipiński et al., 2022). In hematology, pathological liquid phase separation acts as a critical nucleation precursor that accelerates the aberrant polymerization and fibrillation of sickle cell hemoglobin (HbS), thereby driving the core pathology of erythrocyte rigidification and vaso occlusion (Galkin et al., 2002). In oncology, mutant p53 undergoes nuclear LLPS, forming prion like condensates that solidify into amyloid aggregates and facilitate gain of function tumorigenesis (Petronilho et al., 2021). Moreover, oligomerization may lower nucleation barriers and reduce saturation thresholds (Dao et al., 2018). Although the resulting inclusions can transiently buffer toxic oligomers and exert a short lived neuroprotective effect, sustained proteostasis stress or failure of quality control pathways ultimately drives their maturation into fibrillar deposits that perpetuate aggregation and toxicity (Alberti and Dormann, 2019; Tompkins and Hill, 1997). This dynamic highlights a central conceptual tension in the field: the dual role of phase transitions as both adaptive mechanisms and drivers of pathology. Critically, it raises the fundamental question of when and how a physiological response shifts toward becoming pathological.

##### 2.5.4.3 Small-molecule modulation of biomolecular condensates: Mechanisms and therapeutic potential

The internal microenvironment of biomolecular condensates is formed through dynamic interactions among proteins, RNA, and solutes. These interactions govern key condensate properties—such as biochemical reactivity, molecular sorting, and material exchange—and enable the selective recruitment of specific small molecules, which in turn influence condensate stability and assembly. Mechanistically, small molecules can alter the physical state of condensates by modulating their internal environment. Hydrophilic compounds, for example, raise polarity and lower viscosity, while “proteophilic” molecules like ATP exclude water, thereby increasing hydrophobicity and viscosity. Such effects suggest that cells can fine-tune the material state of membraneless organelles through changes in the concentration of endogenous regulators (Pan et al., 2025). However, under pathological conditions, small molecules may act as drivers that promote the conversion of liquid-like condensates into gel-like or solid states (Pan et al., 2025). Studies using purified α-synuclein have further revealed how small molecules can selectively modulate phase separation. Polyphenols such as quercetin and EGCG promote the formation of liquid condensates while potently inhibiting their conversion into solid amyloid fibrils. These molecules bind α-synuclein *via* hydrophobic interactions, enhancing its effective multivalency to drive phase separation, while simultaneously stabilizing conformations that hinder β-sheet assembly required for fibrillation. In contrast, dyes like thioflavin T act as potent inducers that rapidly initiate phase separation and accelerate aggregation (Sarkar et al., 2025). These findings illustrate that small molecules can precisely control the dynamics and phase states of biomolecular condensates, providing a rationale for interfering with pathological protein aggregation. This insight underpins the emerging therapeutic strategy termed Condensate-Modifying Therapeutics (C-mods). Rather than targeting classic protein active sites, C-mods are rationally designed small molecules that directly regulate the assembly, material state, and function of biomolecular condensates—often by tuning properties such as hydrophilicity/hydrophobicity, charge distribution, and three-dimensional conformation (Usman et al., 2025; Wang X. et al., 2024). C-mod activity is highly context-dependent. For instance, polar aliphatic molecules like 1,6-hexanediol can dissolve condensates (e.g., Cajal bodies, stress granules) by disrupting weak hydrophobic interactions, yet under certain conditions they can also promote condensate formation or chromatin compaction through competitive interference with multivalent interactions (Hamad et al., 2021; Trnka et al., 2021). Polar aromatic C-mods frequently operate by weakening inter-chain hydrogen bonds within condensates or *via* competitive binding, thereby preventing protein oligomerization or abnormal conformational transitions—offering new intervention routes for neurodegenerative diseases, cancer, and viral infections (Usman et al., 2025). Beyond synthetic compounds, ions and endogenous metabolites also exhibit dual regulatory capacities. In viral infection, Zn^2+^ promotes phase separation of the viral nucleocapsid protein (N), supporting replication and RNA packaging. Host antiviral responses can counteract this by upregulating Cu^2+^, which competitively inhibits Zn^2+^, disrupting viral morphology and reducing pathogenicity (Monette and Mouland, 2020). Similarly, ATP acts as a key endogenous modulator: at low concentrations it generally enhances phase separation, whereas high concentrations inhibit it. The adenine ring engages in π-π stacking or hydrophobic interactions with protein regions, while the triphosphate chain influences solvent environment; age-related declines in ATP levels compromise the reversibility of phase separation (Meyer and Voigt, 2017; Rice and Rosen, 2017). In summary, C-mods regulate liquid–liquid phase separation through multiple, reversible, and context-sensitive mechanisms. This approach moves beyond the classical “lock-and-key” drug-design model, opening new avenues for treating condensate-related pathologies.

##### 2.5.4.4 Future perspectives

The study of biomolecular condensates is evolving from descriptive observation to quantitative prediction, yet significant challenges persist. Current techniques lack sufficient resolution to dynamically probe condensate organization in living systems, necessitating advanced methods that can precisely manipulate key physicochemical parameters. Fundamental questions remain about cellular control over phase transitions, requiring integrated theoretical frameworks combining Flory-Huggins and active matter principles. Therapeutically, while “molecular glues” and “wedges” offer promising approaches for modulating pathological condensates, achieving specificity presents substantial hurdles. Ultimately, tackling these challenges will demand intensified cross-disciplinary efforts to translate mechanistic insights into clinical applications.
